# Sea urchin-like microstructure pressure sensors with an ultra-broad range and high sensitivity

**DOI:** 10.1038/s41467-021-21958-y

**Published:** 2021-03-19

**Authors:** Xiu-man Wang, Lu-qi Tao, Min Yuan, Ze-ping Wang, Jiabing Yu, Dingli Xie, Feng Luo, Xianping Chen, ChingPing Wong

**Affiliations:** 1 Key Laboratory of Optoelectronic Technology & Systems, Education Ministry of China, Chongqing University and College of Optoelectronic Engineering, Chongqing University, Chongqing, China; 2 State Key Laboratory of Power Transmission Equipment & System Security and New Technology and School of Electrical Engineering, Chongqing University, Chongqing, China; 3 School of Materials Science and Engineering, Georgia Institute of Technology, Atlanta, GA USA

**Keywords:** Sensors and biosensors, Nanoparticles, Design, synthesis and processing

## Abstract

Sensitivity and pressure range are two significant parameters of pressure sensors. Existing pressure sensors have difficulty achieving both high sensitivity and a wide pressure range. Therefore, we propose a new pressure sensor with a ternary nanocomposite Fe_2_O_3_/C@SnO_2_. The sea urchin-like Fe_2_O_3_ structure promotes signal transduction and protects Fe_2_O_3_ needles from mechanical breaking, while the acetylene carbon black improves the conductivity of Fe_2_O_3_. Moreover, one part of the SnO_2_ nanoparticles adheres to the surfaces of Fe_2_O_3_ needles and forms Fe_2_O_3_/SnO_2_ heterostructures, while its other part disperses into the carbon layer to form SnO_2_@C structure. Collectively, the synergistic effects of the three structures (Fe_2_O_3_/C, Fe_2_O_3_/SnO_2_ and SnO_2_@C) improves on the limited pressure response range of a single structure. The experimental results demonstrate that the Fe_2_O_3_/C@SnO_2_ pressure sensor exhibits high sensitivity (680 kPa^−1^), fast response (10 ms), broad range (up to 150 kPa), and good reproducibility (over 3500 cycles under a pressure of 110 kPa), implying that the new pressure sensor has wide application prospects especially in wearable electronic devices and health monitoring.

## Abstract (web summary)

Pressure sensors with high sensitivity and large pressure range is crucial to their various applications in electronic engineering. Here, Wang et al. propose a new design based on a ternary nanocomposite material and show high pressure sensitivity of 680 kPa^−1^ and fast response of 10 ms up to 150 kPa.

## Introduction

In the past few years, pressure sensors are considered promising candidates for use in wearable devices, electronic skins, and human-machine interfaces due to their low cost, flexibility, simple fabrication process, high integration potential, among others^1–4^. In particular, pressure sensors are classified into four main types: capacitive^5–7^, piezoresistive^8–10^, piezoelectric^11–13^, and triboelectric sensors^14–16^. Piezoresistive pressure sensors have multiple benefits, including low energy consumption, easy signal collection, simple device assembly, and high sensitivity. Recently, different microstructure or nanostructure geometries such as (interlocked microstructures^17^, hollow-sphere microstructure^18^, micropyramid array^19^, and porous structure^20^) have been explored to improve the sensitivity of piezoresistive pressure sensors. Among them, the tapering geometry or spine structure confers a clever design that not only promotes signal transduction for high sensitivity, but also protects the bristle from mechanical breaking^21,22^. Similar structures have been used in mechanical sensors and have been shown to improve sensing performance. For example, Yin et al. reported that ZnO sea-urchin-shaped microparticles with a low-temperature solution process exhibited a high sensitivity of 121 kPa^−1^ (pressure range 0–10 Pa)^23^. Lee et al. achieved a sensitivity of 2.46 kPa^−1^ (pressure range 0–1 kPa) with a piezoresistive pressure sensor based on sea-urchin-shaped metal nanoparticles^24^. Furthermore, Shi et al. studied the urchin-like hollow carbon spheres, and reported that the sensitivity reached 260.3 kPa^−1^ at 1 Pa^25^. Therefore, the piezoresistive pressure sensors have a high sensitivity, but only under a small pressure range. Additionally, without any additives, relying only on the structure and performance of conductor and semiconductor, it is difficult to achieve a high sensitivity and a wide pressure working range at the same time.

High sensitivity can be achieved with two conditions; low initial current and large output current changes under a certain pressure^26^. The conductivity of semiconductor is considerably low, so the initial current could be achieved at low level. In addition, the semiconductor/conductor interface piezoresistive effect is favorable for the change of contact area, which leads to a high output current change^26^. A depletion region and band bending occurs in the contact sections of the heterojunction, which induces the lower interfacial resistance and promotes the charge transport/transfer^27^. Heterojunctions have been used in many modern devices, including light emitting diodes (LEDs), photodetectors and solar cells^28–30^. Therefore, when the metal oxide semiconductor/C composite structure and the heterostructures are used in the pressure sensor, the sensing performance of the pressure sensor may be improved.

In this work, we propose a pressure sensor with nanostructure design of materials with contains metal oxide semiconductor/C composite structure and a heterostructure. When fabricated using the new nanostructure, the pressure sensor exhibited ultrasensitivity and an ultra-broad-range. We chose Fe_2_O_3_ and SnO_2_ as sensing materials, because of their low cost, environmental friendliness, and natural abundance. Sea-urchin-like Fe_2_O_3_ was synthesized through a hydrothermal method. This strategy involves the use of acetylene black carbon as a carrier, due to its strong conductivity and high specific surface. One part of acetylene black carbon encloses Fe_2_O_3_ particles, whereas the carbon materials part was embedded in the Fe_2_O_3_ needles gap, forming a Fe_2_O_3_/C structure. Furthermore, one part of the SnO_2_ nanoparticles was dispersed into the carbon layer to form the SnO_2_@C structures, whereas its other part adhered to the Fe_2_O_3_ needles surface to form the Fe_2_O_3_/SnO_2_ heterostructure. Carbon improves the conductivity of a single metal oxide. Collectively, the synergistic effects of the three structures (Fe_2_O_3_/C, Fe_2_O_3_/SnO_2_ and SnO_2_@C) improved the limited pressure response range of a single structure. Notably, the Fe_2_O_3_/C@SnO_2_ (3:1:4) pressure sensor exhibited a high sensitivity (680 kPa^−1^), fast response (10 ms), broad range (up to 150 kPa) and good reproducibility (over 3500 cycles under a pressure of 110 kPa).

## Results

### Structural characterization

**Fig. 1:**
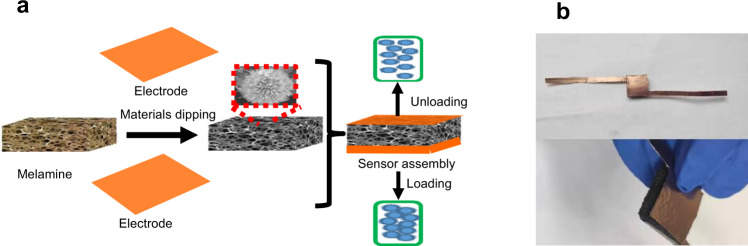
Preparation diagram and sensor images. **a** Schematic illustration of the fabrication of the pressure sensor. **b** Images of the pressure sensor encapsulated with a copper tape.

The fabrication process used in this study is shown in Fig. 1. First, conductive materials were synthesized through a hydrothermal method. Then, a clean melamine sponge was soaked in the sample solution. After the electrode connection, a pressure sensor with a melamine sponge substrate was obtained. Figure 1b is an image of the pressure sensor.

**Fig. 2:**
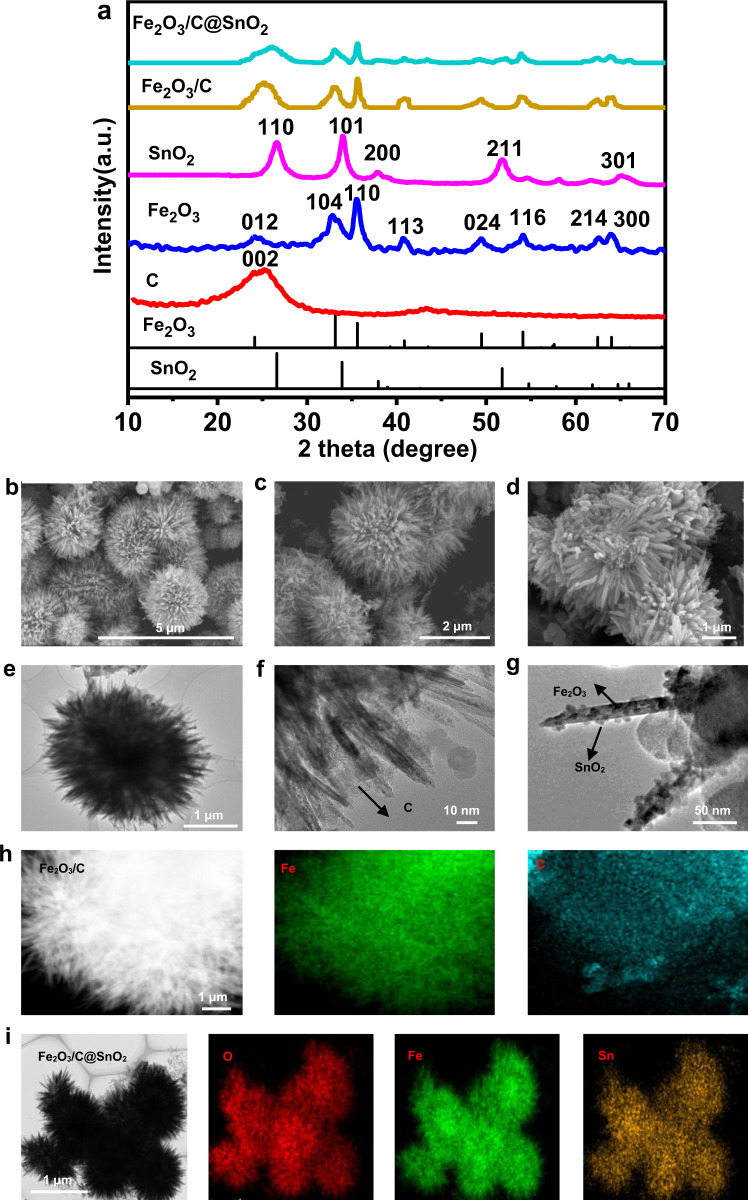
Fe_2_O_3_, SnO_2_, Fe_2_O_3_/C (3:1), and Fe_2_O_3_/C@SnO_2_ (3:1:4) microparticles structure and morphology. **a** The XRD patterns of acetylene carbon black, Fe_2_O_3_, SnO_2_, Fe_2_O_3_/C (3:1) and Fe_2_O_3_/C@SnO_2_ (3:1:4). SEM images of **b** Fe_2_O_3_, **c** Fe_2_O_3_/C (3:1), and **d** Fe_2_O_3_/C@SnO_2_ (3:1:4), TEM images of **e** Fe_2_O_3_, **f** Fe_2_O_3_/C (3:1), and **g** Fe_2_O_3_/C@SnO_2_ (3:1:4), elemental mapping of **h** Fe_2_O_3_/C (3:1), and **i** Fe_2_O_3_/C@SnO_2_(3:1:4).

The phase structures of acetylene carbon black, Fe_2_O_3_, SnO_2_, Fe_2_O_3_/C (the mass ratio of Fe_2_O_3_/C 3:1), and Fe_2_O_3_/C@SnO_2_ (the mass ratio of Fe_2_O_3_/C@SnO_2_ 3:1:4) were characterized by X-ray diffraction (XRD), as shown in Fig. 2a. The acetylene carbon black revealed a broad peak at 20°–30°, corresponding to its (002) crystal plane. The XRD pattern with (012), (104), (110), (113), (024), (116), (214), and (300) was regarded as the formation of Fe_2_O_3_ (JCPDS 33-0664). The pristine SnO_2_ nanoparticles exhibited a tetragonal structure (JCPDS 41-1445). Remarkably, the diffraction peaks of acetylene carbon black and Fe_2_O_3_ were observed in Fe_2_O_3_/C. Besides, as the carbon mass increased, the carbon peaks became stronger (Supplementary Fig. 1a). The XRD pattern of Fe_2_O_3_/C@SnO_2_ exhibited broad peaks. All the diffraction peaks corresponded with single acetylene carbon black, Fe_2_O_3_ and SnO_2_, indicating the Fe_2_O_3_/C@SnO_2_ nanocomposite has a high purity. Notably, the peak of SnO_2_ was stronger in the Fe_2_O_3_/C@SnO_2_ (3:1:8) nanocomposites, implying a higher content of SnO_2_ in this composite (Supplementary Fig. 1b).

The microstructures of the Fe_2_O_3_, Fe_2_O_3_/C (3:1), and Fe${\,\!}_{2}$O${\,\!}_{3}$/C@SnO${\,\!}_{2}$ (3:1:4) were characterized by scanning electron microscopy (SEM), transmission electron microscopy (TEM), elemental mapping, and high-angle annular dark-field scanning transmission electron microscopy (HAADF-STEM) (Fig. 2b–i). The images of Fe_2_O_3_ reflect a typical sea-urchin-like structure, with a diameter of about 3 μm, as shown in Fig. 2b. Microstructures of the Fe_2_O_3_/C nanocomposites are shown in Fig. 2c, f. In addition, one part of acetylene black carbon encloses Fe_2_O_3_ particles, while the other part of carbon materials was embedded in the gap of Fe_2_O_3_ needles, thereby forming a Fe_2_O_3_/C structure. The SEM images of resulting the Fe_2_O_3_/C@SnO_2_ (3:1:4), in which SnO_2_ nanoparticles are visible, as shown in Fig. 2g. Moreover, one part of the SnO_2_ nanoparticles was tightly attached to Fe_2_O_3_ needles, implying the formation of a heterojunction between Fe_2_O_3_ and SnO_2_. Supplementary Fig. 2i shows the interfacial structure of the Fe_2_O_3_/C@SnO_2_ (3:1:4), as revealed by a high-resolution TEM (HRTEM) technique. The SnO_2_ nanoparticles grew on the (104) surface of the Fe_2_O_3_, along the direction of (110) SnO_2_, forming a (104) _Fe_${\,\!}_{2}$_O_${\,\!}_{3}$/ (110) _SnO_${\,\!}_{2}$ heterojunction. Moreover, the other part of SnO_2_ nanoparticles penetrated the carbon layer, forming SnO_2_@/C (Fig. 2g). It was noted that the Fe_2_O_3_/C@SnO_2_ had three structures with Fe_2_O_3_/C, SnO_2_@/C, and Fe_2_O_3_/SnO_2_ heterojunction. As the mass of carbon and SnO_2_ increased, the carbon layer and SnO_2_ nanoparticles also became clear in the SEM images (Supplementary Fig. 2a-f), consistent with the XRD experimental results. Microstructures and compositional distribution of nanocrystals were further determined by STEM and EDX mapping, respectively. The tin signal for SnO_2_, iron signals for Fe_2_O_3_, and carbon signals were overlapped completely across the entire sample, implying that Fe_2_O_3_, carbon, and SnO_2_ were uniformly combined (Fig. 2h, i). The microstructures of the melamine sponge, and Fe_2_O_3_/C@SnO_2_ (3:1:4)/melamine sponge were characterized by SEM. The melamine sponge was found to have a porous and cellular-like structure with interconnected tetrapod-shaped frameworks. The frameworks width was about 4.3 μm (Supplementary Fig. 3a, b). The Fe_2_O_3_/C@SnO_2_/ melamine sponge sample maintained the porous and interconnected structure. Due to the coated Fe_2_O_3_/C@SnO_2_ layer, the surface of the sponge was found to be slightly rough, the framework width was about 4.30–7.34 μm while the thickness of sensing layer was about 0–3.04 μm (Supplementary Fig. 3d).

**Fig. 3:**
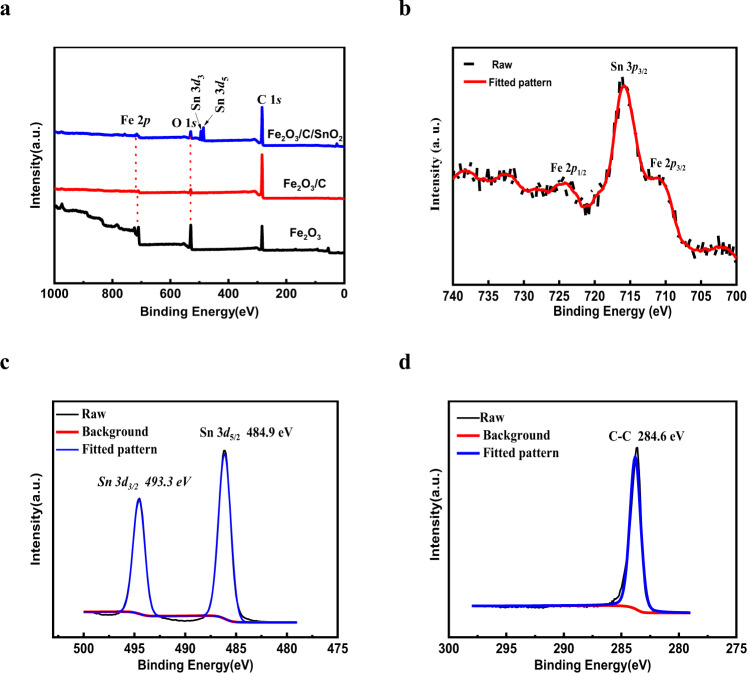
XPS spectrum. **a** The XPS of Fe_2_O_3_, Fe_2_O_3_/C (the mass ratio of 3:1), Fe_2_O_3_/C@SnO_2_ (the mass ratio of 3:1:4), **b** high-resolution curves of **c** Fe, Sn, and **d** C.

Furthermore, to evaluate the composition of Fe_2_O_3_/C and Fe_2_O_3_/C@SnO_2_ nanostructures, the XPS technique was used. The full spectrum characteristic peaks were composed of Fe 2*p*, C 1 *s*, O 1 *s*, and Sn 3*d* states, as displayed in Fig. 3a. The peaks at 712.2 and 725.6 eV were ascribed to the Fe 2*p*_3/2_ and Fe 2*p*_1/2_, while the peak at 715.9 eV was attributed to Sn 3*p*_3/2_^31^. The Fe_2_O_3_/C@SnO_2_ sample shows the Sn 3*d*_3/2_ and Sn 3*d*_5/2_ at around 493.3 and 484.9 eV, with a spin-orbit splitting of 8.4 eV, in concordance with the previously reported energy values for SnO_2_^32^.

### Sensing properties of the Fe_2_O_3_/C@SnO_2_ pressure sensor

**Fig. 4:**
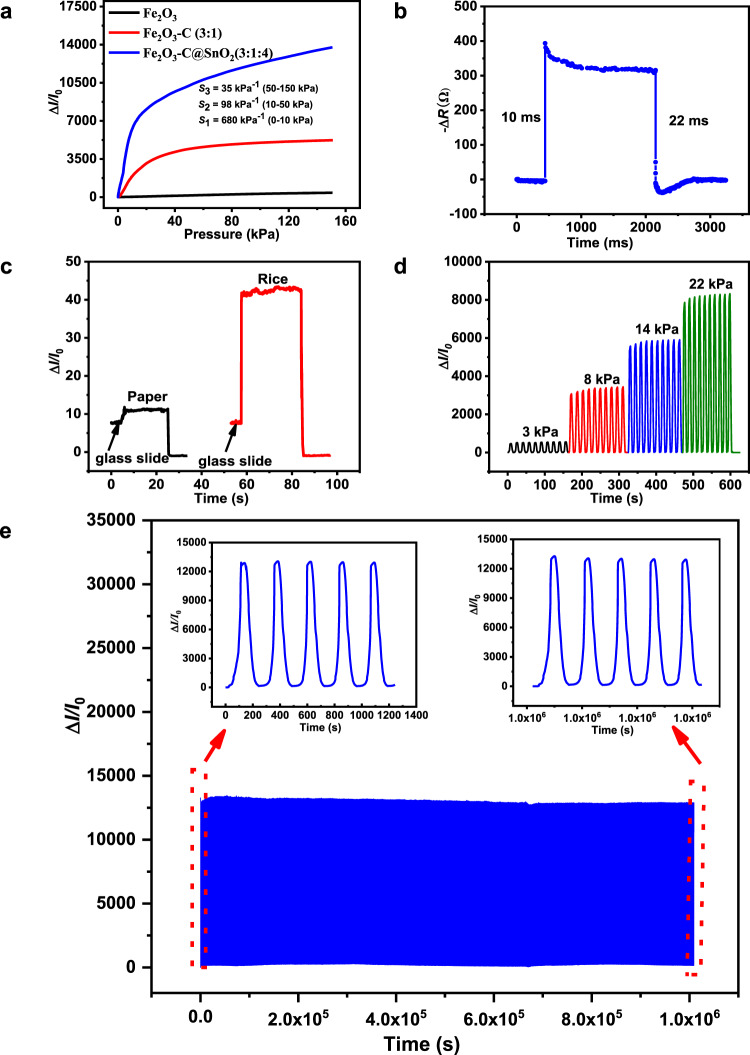
Pressure-sensing characterizations. **a** The sensitivity of Fe_2_O_3_, Fe_2_O_3_/C with the mass of ratio of (3:1), and Fe_2_O_3_/C@SnO_2_ with the mass of ratio of 3:1:4 based sensors. **b** Response time of Fe_2_O_3_/C@SnO_2_ (3:1:4) pressure sensor. **c** Detection of low pressure: current curve of the proposed Fe_2_O_3_/C@SnO_2_ (3:1:4) pressure sensor pressed by paper and rice grain. **d** The current response due to increased pressures under loading and unloading. **e** Stability performance of the Fe_2_O_3_/C@SnO_2_ (3:1:4) pressure sensor with loading-unloading of more than 3500 cycles.

To measure the piezoresistive characteristics of the pressure sensors, we setup a custom-made system composed of a universal testing machine and a digital source meter. Sensitivity was calculated using the formula: *S* = (Δ*I*/*I*_unloading_)/Δ*p*, where Δ*I* = *I*_loading_ − *I*_unloading_, represents the relative current change, Δ*p* refers to the change of pressure. Figure 4a, Supplementary Fig. 4, and Supplementary Table. 1 show the obtained measurements. The sensitivity of Fe_2_O_3_/C@SnO_2_ pressure sensor was higher than that of Fe_2_O_3_, Fe_2_O_3_/C, and SnO_2_@C pressure sensors. The sensitivity of Fe_2_O_3_/C@SnO_2_ (3:1:4) sensor was *S*_1_ ~ 680 kPa^−1^ when the pressure was below 10 kPa, *S*_2_ ~ 98 kPa^−1^ when the pressure was ranged from 10 to 50 kPa, and *S*_3_ ~ 35 kPa^−1^ when the pressure was ranged from 50 to 150 kPa. This sensitivity is higher than that of ZnO sea-urchin-like, carbon sea-urchin-like, Ag/Au sea-urchin-like, and other pressure sensors (Supplementary Table 2). Even through the sea-urchin-like Fe_2_O_3_ structure promoted signal transduction and protected the Fe_2_O_3_ needles from mechanical breakage, the sensitivity of the sensor (3 kPa^−1^) was still low, because Fe_2_O_3_ has poor conductivity. In addition, current changes were still small even under the high pressure. Supplementary Fig. 4a and Supplementary Table. 1 shows the current response of the pressure sensors under different mass ratios of Fe_2_O_3_ and carbon. The mass ratio of 3:1 exhibited the highest sensitivity (203 kPa^−1^) compared to the other lower ratios (2:1, 1:1 and 1:2) and to the higher one (4:1), let alone the pure carbon. The high sensitivity of the Fe_2_O_3_/C pressure sensor can be attributed to several factors. The microfibers of sponge are composed of nanocomposites, therefore, as the contact area is increased, there is corresponding an increase of current (Supplementary Fig. 12). Moreover, when carbon is added into the Fe_2_O_3_ system, the Fe_2_O_3_/C nanocomposite exhibits a larger current variation compared to that of pure carbon. Under the constant mass of Fe_2_O_3_, and the increased amounts of carbon, the sensitivity of the Fe_2_O_3_/C pressure sensor increases due to an increase of the conductive path. However, excessive addition of carbon to the sensor may significantly increase conductivity (when the mass ratio of carbon and Fe_2_O_3_ exceeds 1:3), thus making it a good conductor, and in return affecting its increase in the corresponding conductive pathways. Here, we noted that the addition of carbon greatly improved the sensitivity of the sensor in the pressure range within 50 kPa, but did not improve the sensitivity in the pressure range over 50 kPa (Supplementary Fig. 4a).

To improve the sensitivity of the pressure sensor under a high-pressure range (over 50 kPa), Fe_2_O_3_/C (3:1) was further combined with SnO_2_. The addition of SnO_2_ nanoparticles improve the sensitivity of the sensor in the low-pressure range (within 50 kPa) and improves its sensitivity in the pressure range (over 50 kPa) (Supplementary Fig. 4b and Supplementary Table. 1). Furthermore, when the two semiconductors were brought in contact and subjected to high-temperature calcination, the band alignment occurs driven by the equilibration of the Fermi level (Supplementary Fig. 10)^33,34^. Consequently, an n-n type heterostructure was formed between Fe_2_O_3_ and SnO_2_, which promoted the transfer of electrons from Fe_2_O_3_ to SnO_2_, thus enhancing the conductivity of the pressure sensor. Supplementary Fig. 2i shows that the SnO_2_ nanoparticles grew on the (104) surface of the Fe_2_O_3_, along the direction of (110) SnO_2_, thereby a forming (104) _Fe_${\,\!}_{2}$_O_${\,\!}_{3}$/ (110) _SnO_${\,\!}_{2}$ heterojunction.

To further prove that the formation of heterojunction in Fe_2_O_3_/C@SnO_2_ nanocomposites improved the sensing performance of the Fe_2_O_3_/C@SnO_2_ pressure sensor. The sensing performances of the Fe_2_O_3_/SnO_2_(3:4) and Fe_2_O_3_/C@Fe_2_O_3_(3:1:4) pressure sensors were measured (Supplementary Fig. 5a–c). The sensitivity of Fe_2_O_3_/SnO_2_ (3:4) sensor was *S*_1_ ~ 8.5 kPa^−1^ when the pressure was below 10 kPa, *S*_2_ ~ 8.5 kPa^−1^ when the pressure was ranged from 10 to 50 kPa, and *S*_3_ ~ 8 kPa^−1^ when the pressure ranged from 50 to 150 kPa. It was higher than those of single Fe_2_O_3_ (*S*_1_ ~ 3 kPa^−1^, *S*_2_ ~ 3 kPa^−1^, and *S*_3_ ~ 2 kPa^−1^) and single SnO_2_ pressure sensors (*S*_1_ ~ 1 kPa^−1^, *S*_2_ ~ 1 kPa^−1^, and *S*_3_ ~ 0.6 kPa^−1^). In addition, the sensitivity of Fe_2_O_3_/C@Fe_2_O_3_ (3:1:4) pressure sensor was *S*_1_ ~ 70 kPa^−1^ when the pressure was below 10 kPa, *S*_2_ ~ 9 kPa^−1^ when the pressure ranged from 10 to 50 kPa, and *S*_3_ ~ 2 kPa^−1^ when the pressure ranged from 50 to 150 kPa (Supplementary Fig. 5c). The sensitivity of Fe_2_O_3_/C@Fe_2_O_3_ (3:1:4) pressure sensor was lower than that of Fe_2_O_3_/C@SnO_2_ (3:1:4) pressure sensor (*S*_1_ ~ 680 kPa^−1^, *S*_2_ ~ 98 kPa^−1^, and *S*_3_ ~ 35 kPa^−1^) (Supplementary Fig. 5b). The images of Fe_2_O_3_/C@Fe_2_O_3_ (3:1:4) reflect a typical sea-urchin-like structure (Supplementary Fig. 6). These findings show that the Fe_2_O_3_/C@SnO_2_ heterostructure can improve the sensing performance of pressure sensor.

Collectively, the synergistic effects of the three structures (Fe_2_O_3_/C, Fe_2_O_3_/SnO_2_ and SnO_2_@C) improved the limited pressure response range of a single structure. Notably, the content of SnO_2_ in Fe_2_O_3_/C@SnO_2_ was associated with significantly improved the performance of the pressure sensor as shown in Supplementary Fig. 4b. In addition, when more SnO_2_ (Fe_2_O_3_/C@SnO_2_(3:1:8)) was added in the synthesis, the polymerization of SnO_2_ nanoparticles occurred due to their high surface energy, and subsequently leading to a non-uniformed distribution of SnO_2_ nanoparticles in Fe_2_O_3_/C@SnO_2_. However, when less SnO_2_ (Fe_2_O_3_/C@SnO_2_(3:1:1)) was added in the synthesis, less accumulation layer was formed on SnO_2_, thereby affecting the conductivity of the pressure sensor. Therefore, the obtained Fe_2_O_3_/C@SnO_2_(3:1:8) and Fe_2_O_3_/C@SnO_2_(3:1:1) had a lower sensitivity compared to Fe_2_O_3_/C@SnO_2_(3:1:4). Moreover, Fe_2_O_3_/C@Sb_2_O_3_ (3:1:4) was synthesized and characterized to verify whether the ternary structure has a certain universality in improving the sensitivity and expanding the pressure working range of the piezoresistive pressure sensor. The sensitivity of Fe_2_O_3_/C@Sb_2_O_3_ (3:1:4) pressure sensor was found to be *S*_1_ ~ 303 kPa^−1^ when the pressure was below 10 kPa, *S*_2_ ~ 180 kPa^−1^ within the pressure range of 10–50 kPa, and *S*_3_ ~ 13 kPa^−1^ within the pressure range of 50–150 kPa (Supplementary Fig. 11). The experimental results show that the ternary structure has a certain universality in improving the sensitivity and in expanding the pressure working range of the piezoresistive pressure sensor.

To determine whether the sensing performance of the pressure sensor is affected by humidity, we tested the sensing performance of the Fe_2_O_3_/C@SnO_2_ (3:1:4) pressure sensor at room temperature with a relative humidity (RH) of 73%, 85%, and 95%. Supplementary Fig. 7 shows that the sensing performance of the Fe_2_O_3_/C@SnO_2_ (3:1:4) pressure sensors did not change with increasing the relative humidity, implying that their sensing performance was independent of relativity humidity. In addition, variations of current ratios with the pressure of the sponges with different areas and thicknesses was similar (Supplementary Fig. 8a–f), which indicates that the sensing performance of the Fe_2_O_3_/C@SnO_2_ sponge is independent of its area and thickness. All results show that the Fe_2_O_3_/C@SnO_2_ pressure sensors are stable.

Besides, we further assessed the low limit detection of the Fe_2_O_3_/C@SnO_2_ (3:1:4) pressure sensor as outlined in Fig. 4c. To evaluate the low limit detection of the pressure sensor, a weight of 4.2 g glass slide was placed on the pressure sensor. Thereafter, a paper (~0.52 pa, *m* = 0.0107 g, *S* = 1 × 2 cm^2^) and rice (*m* = 0.0116 g) were put on the glass slide. The glass slide served two purposes; completing the contact between the electrode and the pressure sensor, and stabilizing the current more. The response time of the Fe_2_O_3_/C@SnO_2_ (3:1:4) pressure sensor is shown in Fig. 4b. When compressing the sponge pressure sensor by 0.02 mm at a speed of 500 mm per min, the response time and recovery time of the Fe_2_O_3_/C@SnO_2_ (3:1:4) pressure sensor was 10 and 22 ms, respectively. The hysteresis of the recovery time of this pressure sensor may be attributed to the sponge substrate. To evaluate the stability of the pressure sensor under different pressures, the Fe_2_O_3_/C@SnO_2_ (3:1:4) pressure sensor was set under various pressure values of 3, 8, 14, and 22 kPa (Fig. 4d). These findings reveal that the current gradually increases with increasing pressure. Therefore, the Fe_2_O_3_/C@SnO_2_ (3:1:4) pressure sensor can clearly distinguish different pressure values. Furthermore, repeated compression/release test over 3500 cycles with a peak pressure of 110 kPa was performed (Fig. 4e). The insets revealed the 5 cycles of the current response at the inception (left) and termination (right) of the stability test, while, the device indicated a stable signal without offset during the cycles test, thereby reflecting that the performance of the sensor was stable under long cycles and high pressure. The SEM images of sponge in the original state and compression/release over 3500 cycles state are shown in Supplementary Fig. 2g, h. Compared to the original state, the micromorphology of Fe_2_O_3_ did not exhibit any change when the sensor was repeatedly compressed/released under high pressure. However, only a small part of the Fe_2_O_3_ needles was detached from the sea-urchin-shaped microspheres. In this work, the good stability of the sensor could be attributed to two reasons; the tapering geometry of Fe_2_O_3_ protects the bristle from mechanical breaking^21,22^, and there is a lot of space between the Fe_2_O_3_ needle, which can allow carbon and SnO_2_ nanoparticles to easily absorb onto the interval gap, thereby protecting the integrity of the Fe_2_O_3_ needle structure.

### Extremely high-pressure resolution

**Fig. 5:**
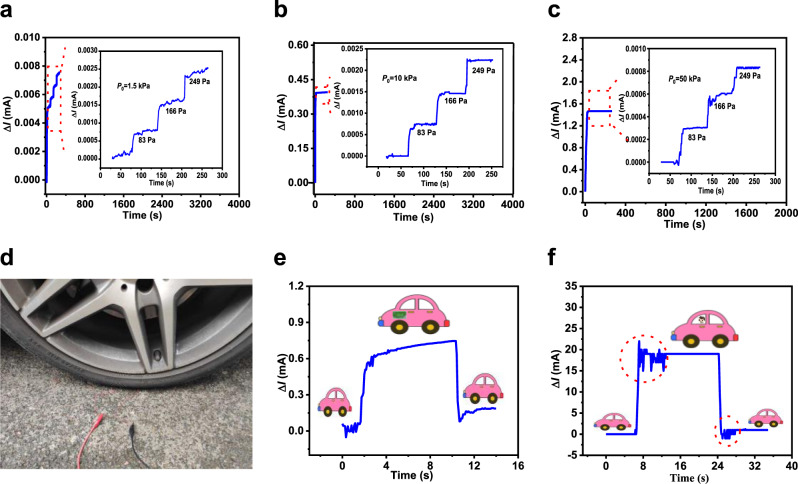
Extremely high-sensing resolution of the Fe_2_O_3_/C@SnO_2_ pressure sensor. Detection of micro pressure under loading pressures of **a** 1.5 kPa, **b** 10 kPa, and **c** 50 kPa. **d** Experimental setup of a car with a Fe_2_O_3_/C@SnO_2_ (3:1:4) pressure sensor attached under a front tire. **e** Current signals corresponding to an unloaded, loaded, and unloaded 4 kg carton of milk on the driving seat of the car. **f** Current signals corresponding to a 73 kg male passenger getting into and out of the car.

A key feature of the Fe_2_O_3_/C@SnO_2_ (3:1:4) pressure sensor is high sensitivity in wide pressure ranges. Therefore, we evaluated the sensitivity of this pressure sensor under high pressure by subjecting it to different pressure values at 1.5, 10 and 50 kPa (Fig. 5a–c). First, the pressure sensor was subjected to the set pressure value, followed by consecutive addition of three coins, each weighing about 3.19 g, equivalent to a pressure of 86 Pa. Each pressure increment caused a stepwise increment in current, with the current signal being stable. In another experiment, a pressure sensor with a volume of *V* = 19 × 19 × 4 mm^3^ was placed under the front wheel of a car (the weight of the car was 1670 kg) as shown in Fig. 5d. Thereafter, a carton of milk weighing 4 kg put on the driving seat of the car and then taken away as indicated in Fig. 5e. The changes in current were successfully detected. When the male passenger with a weight of 73 kg gets into or out of the car, the current changed significantly (Fig. 5f). The circled vibrations in Fig. 5f reveal that the pressure sensor can accurately capture the movement of the male passenger getting on or off the car. The sensitivity of the pressure sensor under high pressure was also tested by the tensile test equipment. The sensing performance of device under a pressure of *P*_0_ = 210 kPa is shown in Supplementary Fig. 9a, b. During the test, the device was first subjected to the reference pressure. Then, the pressure sensor was added with pressure of 2.8 and 25 kPa, respectively. It was found that the pressure sensor still had high sensitivity under the high pressure.

### Wearable device demonstration

**Fig. 6:**
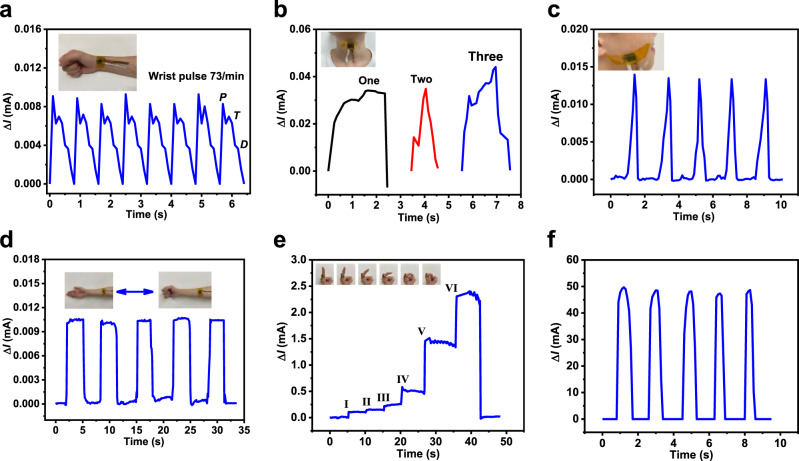
Wearable demonstration. **a** The current response caused by the arterial pulse waves with the sensor attached to the wrist. **b** The recorded current signal versus time pronouncing. Finally, **c** the signal variations of relative current corresponding to different occlusion, **d** human palm, **e** finger motion, and **f** walking.

Due to the high sensitivity, fast response time, and broad pressure regime of the pressure sensor (Fe_2_O_3_/C@SnO_2_ (3:1:4)), it can be applied in various fields. For instance, it can be used to detect voice, wrist pulse, and human motion activities. The Fe_2_O_3_/C@SnO_2_ (3:1:4) pressure sensor was attached to the skin with the help of polyimide (PI) tape for all human body interactions. Figure 6a shows the real-time wrist pulse as detected using the Fe_2_O_3_/C@SnO_2_ (3:1:4) pressure sensor. The testing curves revealed strong characteristic peaks of the human sphygmic waveforms, with the pulse rate being about 73 times per min, which is the normal level. Based on the excellent performance of the Fe_2_O_3_/C@SnO_2_ (3:1:4) pressure sensor, it can be used for monitoring human health. This pressure sensor was also attached to the human throat to monitor and distinguish subtle differences of muscle motions near the throat, when the words one, two and three were spoken (Fig. 6b). Interestingly, this technique can be used by deaf and mute people who are unable to speak. It is well-known that their vocal cords can vibrate, and thus, the vibration produced can be transformed into the required sound^7^. Moreover, the pressure sensor was mounted on the cheek to monitor the occlusion movements of humans, as displayed in Fig. 6c. Upon occlusion, the current was found to have significantly changed, proving the excellent performance of the sensor. Moreover, the pressure sensor was also attached to the arm to detect radial muscle contractions, which occur when making a fist (Fig. 6d). When the tester made a fist, the current signal and compression of the pressure sensor increased, illustrating its potential application in physical training and curing muscle damage. The response of the pressure sensor for continuously bending six different motions of the finger is presented in Fig. 6e. It showed different responsive current signals for different motions of the finger. In particular, the current signal exhibited a slight increase when the finger bent in small-scale (motion-I, motion-II, and motion-III), whereas larger-scale bending led to a sharp increase of current value (motion-IV, motion-V, and motion-VI). Large-scale movements resulted in a strong compression of the pressure sensor, thereby forming more conductive pathways. These findings imply that the pressure sensor can precisely distinguish different-scale motions of the finger. Figure 6f shows that the pressure sensor was also mounted on foot using tape to monitor walking states. The response signals of walking motion were stable and repetitive, suggesting that it can be applied in gait recognition and motion monitoring. These outcomes show that the Fe_2_O_3_/C@SnO_2_ pressure sensor has broad application prospects in medical health and wearable electronic devices.

## Discussion

In summary, we present a sea-urchin-shaped microstructure Fe_2_O_3_/C@SnO_2,_ which was synthesized using a simple and environmentally friendly hydrothermal method. Moreover, we provide a pressure sensor with high sensitivity and a large working range based on a simple dip-coating process method. The Fe_2_O_3_/C@SnO_2_ pressure sensor exhibits high sensitivity (680 kPa^−1^), fast response (10 ms), broad range (up to 150 kPa), and good reproducibility (over 3500 cycles under a pressure of 110 kPa). Interestingly, multiple human physiological activities (such as pulse, pronunciations, joint bending, and walking, among others) could be monitored by the Fe_2_O_3_/C@SnO_2_ pressure sensor. Based on the above excellent performances of this device, it has significant implications in wearable electronics, health monitoring, and measuring pressure distribution.

## Methods

### Synthesis of Fe_2_O_3_

The method used for the synthesis of Fe_2_O_3_ used in this study^35^. In particular, the Fe_2_O_3_ was synthesized through a hydrothermal process, where 0.405 g FeCl_3_.6H_2_O (Aladdin) and 0.205 g Na_2_SO_4_ (Aladdin) were first dissolved in distilled water (30 mL) and stirred for 10 min. Then, the mixture was heated at 120 °C for 6 h in a Teflon-lined stainless-steel autoclave. After cooling, filtering, drying, and thermal annealing at 400 °C for 3 h under air, Fe_2_O_3_ was obtained.

### Preparation of Fe_2_O_3_/C composites

Besides adding different masses of carbon, the same process was used for the synthesis of Fe_2_O_3_/C composites.

### Synthesis of Fe_2_O_3_/C@SnO_2_ composites

First, the Fe_2_O_3_/C composite (0.145 g), Na_2_SnO_3_ (0.145 g, 0.036 g, 0.290 g) (Aladdin), and urea (1.16 g, 0.290 g, 2.320 g) (Aladdin) were dissolved in ethanol/H_2_O solution and kept V_ethanol_: V_H_${\,\!}_{2}$_O_ = 2:3. After stirring for 15 min, the mixture was heated at 180 °C for 6 h in a Teflon-lined stainless-steel autoclave. After natural cooling, the Fe_2_O_3_/C@SnO_2_ composites were washed several times using distilled water and absolute ethanol. Finally, the Fe_2_O_3_/C@SnO_2_ composites were dried at 60 °C for 12 h.

### Synthesis of Fe_2_O_3_/C@Sb_2_O_3_ composites

The Fe_2_O_3_/C@Sb_2_O_3_ composites was synthesized through a hydrothermal process, where 0.34 g Fe_2_O_3_/C and 0.34 g SbCl_3_ (Aladdin) were first dissolved in ethanol (40 mL) and stirred for 30 min. Afterward, the mixture was heated at 140 °C for 12 h in a Teflon-lined stainless-steel autoclave. Lastly, after cooling, filtering, drying, and thermal annealing at 400 °C for 3 h under air, Fe_2_O_3_/C@Sb_2_O_3_ was obtained.

### Preparation of conductive sponges and electrode

A melamine sponge was cut into a cuboid with a length of 19 mm, width (19 mm), and height (4 mm). Then, it was washed several times using ethanol and dried at 45 °C. Thereafter, Fe_2_O_3_/C@SnO_2_ (SnO_2_@C, Fe_2_O_3_/C, Fe_2_O_3_, SnO_2_, and C) and Polyvinylidene fluoride (PVDF) binder (Aladdin) were dissolved in N-methyl pyrrolidone (NMP) (Aladdin) in a weight ratio of 10:1 and mixed to form a slurry. Next, the melamine sponge strip was immersed in the slurry until it was full and dried at 45 °C in a vacuum. A copper wire was then fixed on the polyimide (PI) film (Kapton) substrate coated with laser-induced graphene (LIG) with the silver paste, where the PI-LIG layer acted as the electrode of the device. The copper tape was fixed on the PI film to flat the PI film.

### Structural characterization and the performance of sensors

The crystal structures of the samples were explored using X-ray diffraction (XRD, PANalytical X’Pert Powder), while, the morphology was characterized using scanning electron microscopy (SEM; Quattro S), high-angle annular dark-field scanning transmission electron microscopy (HAADF-STEM), elemental mapping and transmission electron microscopy (TEM; Talos F200S). Binding energy of the products was investigated using X-ray photoelectron spectroscopy (XPS; ESCALAB250Xi). Moreover, the loading of pressure was examined using a universal testing machine (ETM-5038, Shenzhen Wance Testing Machine Co., Ltd.), while the electrical signals of the pressure sensors were recorded at the same time using a digital meter (Keithley 2450, Optoelectronic Technology & Systems) at a constant voltage of 0.1 V. Finally, to assess the response time of the pressure sensor, a multimeter (Keithley DAQ6510, Optoelectronic Technology & Systems) was used.

## Supplementary information

Supplementary Information

Peer Review File

## Data Availability

The data used this study are available from the corresponding author upon reasonable request.

## References

[1] Shin MK (2010). Elastomeric conductive composites based on carbon nanotube forests. Adv. Mater..

[2] Jian M (2017). Flexible and highly sensitive pressure sensors based on bionic hierarchical structures. Adv. Funct. Mater..

[3] Zhou Z, Li Y, Cheng J, Chen S, Li L (2018). Supersensitive all-fabric pressure sensors using printed textile electrode arrays for human motion monitoring and human–machine interaction. J. Mater. Chem. C..

[4] Schwartz G (2013). Flexible polymer transistors with high pressure sensitivity for application in electronic skin and health monitoring. Nat. Commun..

[5] Bai N, Wang L, Wang Q, Deng J, Guo CF (2020). Graded intrafillable architecture-based iontronic pressure sensor with ultra-broad-range high sensitivity. Nat. Commun..

[6] Cho SH (2017). Micropatterned pyramidal ionic gels for sensing broad-range pressures with high sensitivity. ACS Appl. Mater. Interfaces.

[7] Mannsfeld SC (2010). Highly sensitive flexible pressure sensors with microstructured rubber dielectric layers. Nat. Mater..

[8] Pang Y (2016). A flexible highly sensitive and wearable pressure and strain sensors with graphene porous network structure. ACS Appl. Mater. Interfaces.

[9] Maheshwari V, Saraf R (2006). High-resolution thin-film device to sense texture by touch. Science.

[10] Lou Z, Chen S, Wang L, Jiang K, Shen G (2016). An ultra-sensitive and rapid response speed graphene pressure sensor for electronic skin and health monitoring. Nano Energy.

[11] Sun J (2016). Piezo-phototronic effect improved performance of n -ZnO nano-arrays/ p -Cu2O film based pressure sensor synthesized on flexible Cu foil. Nano Energy.

[12] Wen zhuo (2013). Taxel-addressable matrix of vertical-nanowire piezotronic transistors for active and adaptive tactile imaging. Science.

[13] Sumin (2015). Transparent and stretchable interactive human machine interface based on patterned graphene heterostructures. Adv. Funct. Mater..

[14] Khan U (2017). Graphene tribotronics: graphene tribotronics for electronic skin and touch screen applications. Adv. Mater..

[15] Wang X (2016). Self‐powered high‐resolution and pressure‐sensitive triboelectric sensor matrix for real‐time tactile mapping. Adv. Mater..

[16] Su Y, Chen J, Wu Z, Jiang Y (2015). Low temperature dependence of triboelectric effect for energy harvesting and self-powered active sensing. Appl. Phys. Lett..

[17] Pang C (2012). A flexible and highly sensitive strain-gauge sensor using reversible interlocking of nanofibers. Nat. Mater..

[18] Pan L (2014). An ultra-sensitive resistive pressure sensor based on hollow-sphere microstructure induced elasticity in conducting polymer film. Nat. Commun..

[19] Choong CL (2014). Highly stretchable resistive pressure sensors using a conductive elastomeric composite on a micropyramid array. Adv. Mater..

[20] Chen XP (2019). A dual‐functional graphene‐based self‐alarm health‐monitoring e‐skin. Adv. Funct. Mater..

[21] Dechant H-E, Rammerstorfer FG, Barth FG (2001). Arthropod touch reception: stimulus transformation and finite element model of spider tactile hairs. J. Comp. Physiol. A..

[22] Barth FG (2004). Spider mechanoreceptors. Curr. Opin. Neurobiol..

[23] Yin B, Liu X, Gao H, Fu T, Yao J (2018). Bioinspired and bristled microparticles for ultrasensitive pressure and strain sensors. Nat. Commun..

[24] Lee D (2016). Highly sensitive, transparent, and durable pressure sensors based on sea‐urchin shaped metal nanoparticles. Adv. Mater..

[25] Shi L (2020). Quantum effect-based flexible and transparent pressure sensors with ultrahigh sensitivity and sensing density. Nat. Commun..

[26] Wang ZW (2018). The Semiconductor/conductor interface piezoresistive effect in an organic transistor for highly sensitive pressure sensors. Adv. Mater..

[27] Tang HY (2019). Ultra-high sensitive NO_2_ gas sensor based on tunable polarity transport in CVD-WS2/IGZO P-N heterojunction. ACS Appl. Mater. Interfaces.

[28] Park BH (1999). Lanthanum-substituted bismuth titanate for use in non-volatile memories. Nature.

[29] Sze, S. M. & Ng, K. K. *Physics of Semiconductor Devices* (Wiley-Interscience, 2007).

[30] Ashcroft, N. W. & Mermin, N. D. *Solid State Physics* (Saunders College Publishing, 1976).

[31] Wang BB (2014). Fabrication and gas sensing properties of hollow core–shell SnO_2_/α-Fe_2_O_3_. heterogeneous structures. J. Alloy. Compd..

[32] Tian Q, Li L, Chen J, Yang L, Hirano SI (2018). Facile fabrication of robust TiO_2_@SnO_2_@C hollow nanobelts for outstanding lithium storage. J. Power Source.

[33] Zhou X (2018). Ordered porous metal oxide semiconductors for gas sensing. Chin. Chem. Lett..

[34] Zhao T (2019). Hierarchical branched mesoporous TiO_2_–SnO_2_ nanocomposites with well‐defined n–n heterojunctions for highly efficient ethanol sensing. Adv. Sci..

[35] Wang XM (2019). Excellent cyclic performance of Fe_2_O_3_@C/SnO_2_ controlled by Fe_2_O_3_@C and SnO_2_/C hybrid structures for lithium-ion batteries. J. Phys. Chem. Solids.

