# Supplementary material for: Sea urchin-like microstructure pressure sensors with an ultra-broad range and high sensitivity
Source: Nat Commun. 2021 Mar 19;12:1776. doi: 10.1038/s41467-021-21958-y (PMC7979929; doi:10.1038/s41467-021-21958-y)
Supplement: Supplementary file 1 — Supplementary Information [file 41467_2021_21958_MOESM1_ESM.pdf]

Sea urchin-like microstructures pressure sensors with an ultra-broad range and high sensitivity

Xiu-man Wang[1], Lu-qi Tao[2], Min Yuan[1], Zeping Wang[1], Jiabing Yu[1], Dingli Xie[1], Feng Luo[1], Xianping Chen*[1,2] & ChingPing Wong*[3]

[1]Key Laboratory of Optoelectronic Technology & Systems, Education Ministry of China, Chongqing University and College of Optoelectronic Engineering, Chongqing University, Chongqing 400044, China.

[2]State Key Laboratory of Power Transmission Equipment & System Security and New Technology and School of Electrical Engineering, Chongqing University, Chongqing 400044, China.

[3]School of Materials Science and Engineering, Georgia Institute of Technology

***Corresponding author:** xianpingchen@cqu.edu.cn cp.wong@mse.gatech.edu

Supplementary Figures

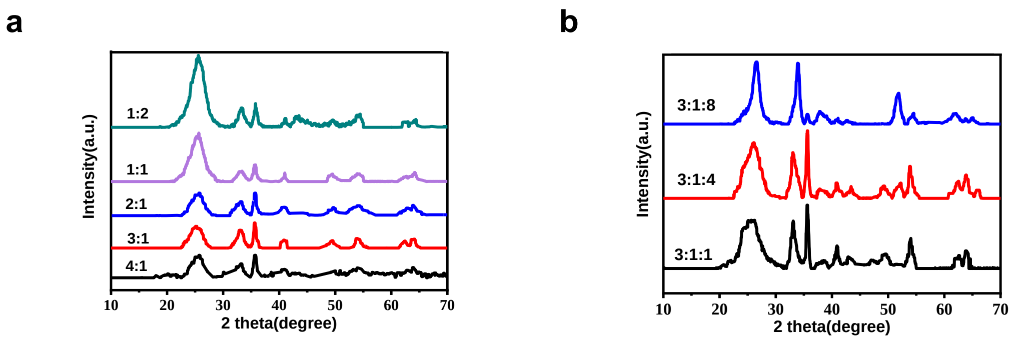

Supplementary Figures 1. XRD patterns. **a** the different mass ratios of Fe_2O_3/C (4:1,3:1,2:1,1:1,1:2), **b** the different mass ratios of Fe_2O_3/C@SnO_2(3:1:1,3:1:4,3:1:8)

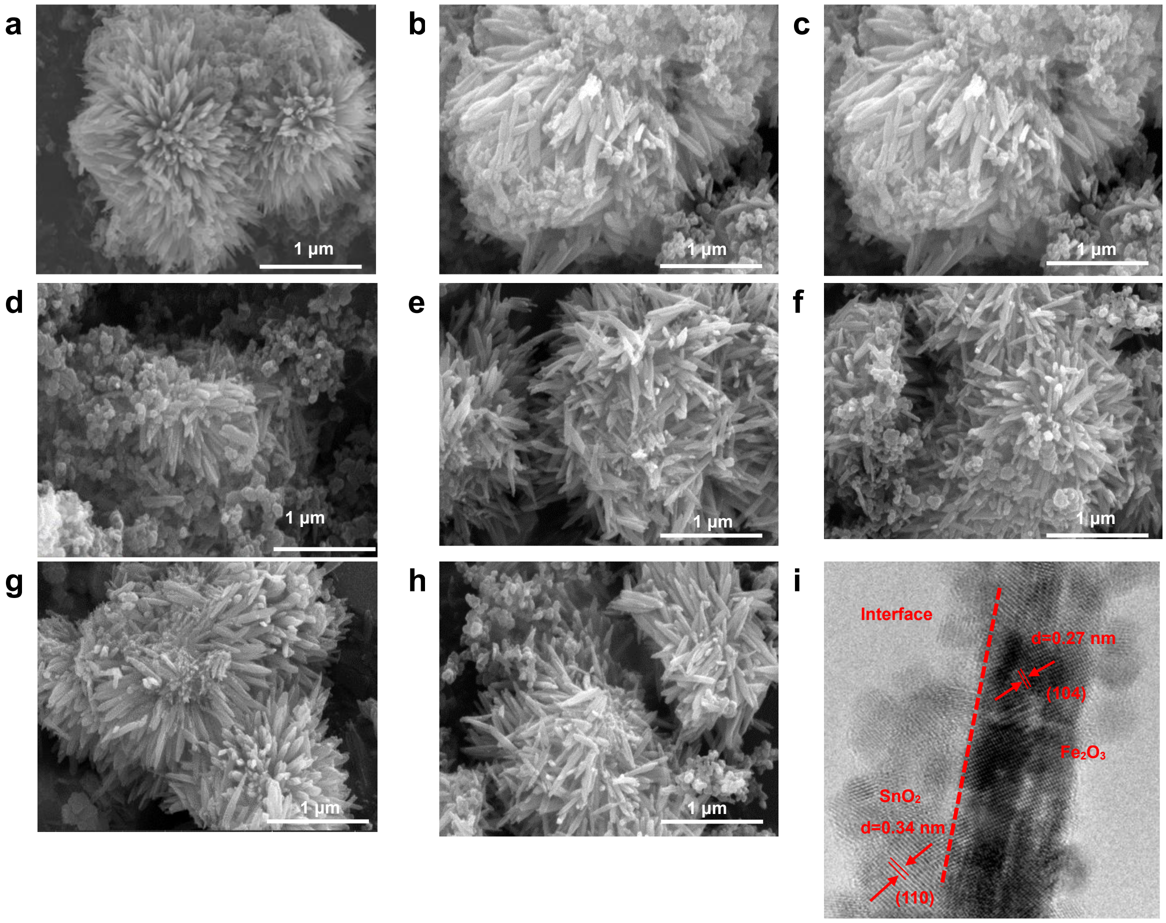

Supplementary Figure 2. Fe_2O_3/C and Fe_2O_3/C@SnO_2 microparticle morphology. The SEM images of the different mass ratios of Fe_2O_3/C (**a** 4:1, **b** 2:1, **c** 1:1, **d** 1:2), the different mass ratios of Fe_2O_3/C@SnO_2 (**e** 3:1:1, **f** 3:1:8, **g** 3:1:4) , **g** and **h** the mass ratio of Fe_2O_3, C, and SnO_2 3:1:4 after loading-unloading for 3500 cycles.**i.** High resolution TEM(HRTEM) images of the mass ratio of Fe_2O_3, C and SnO_2 3:1:4.

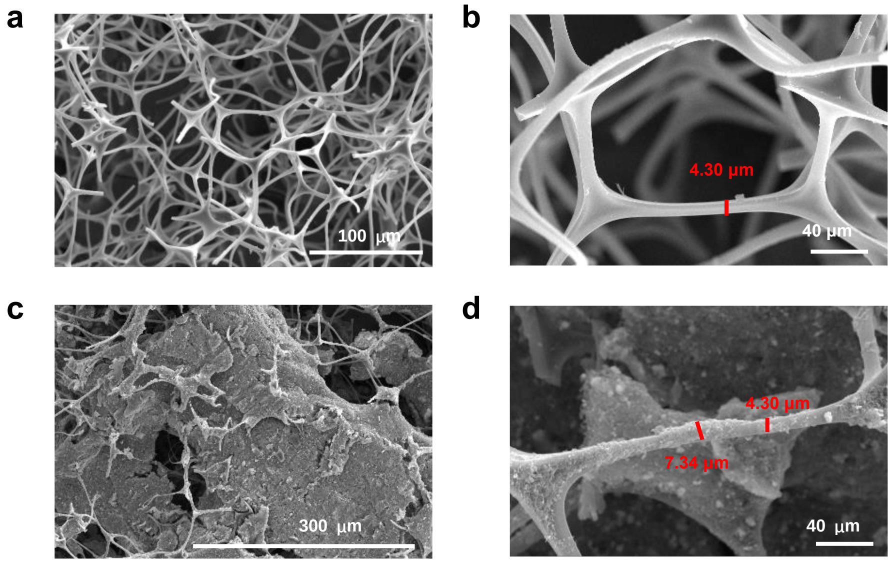

Supplementary Figure 3. SEM images. **a** and **b** SEM images of melamine sponge, **c** and **d** SEM images of Fe_2O_3/C@SnO_2/ melamine sponge.

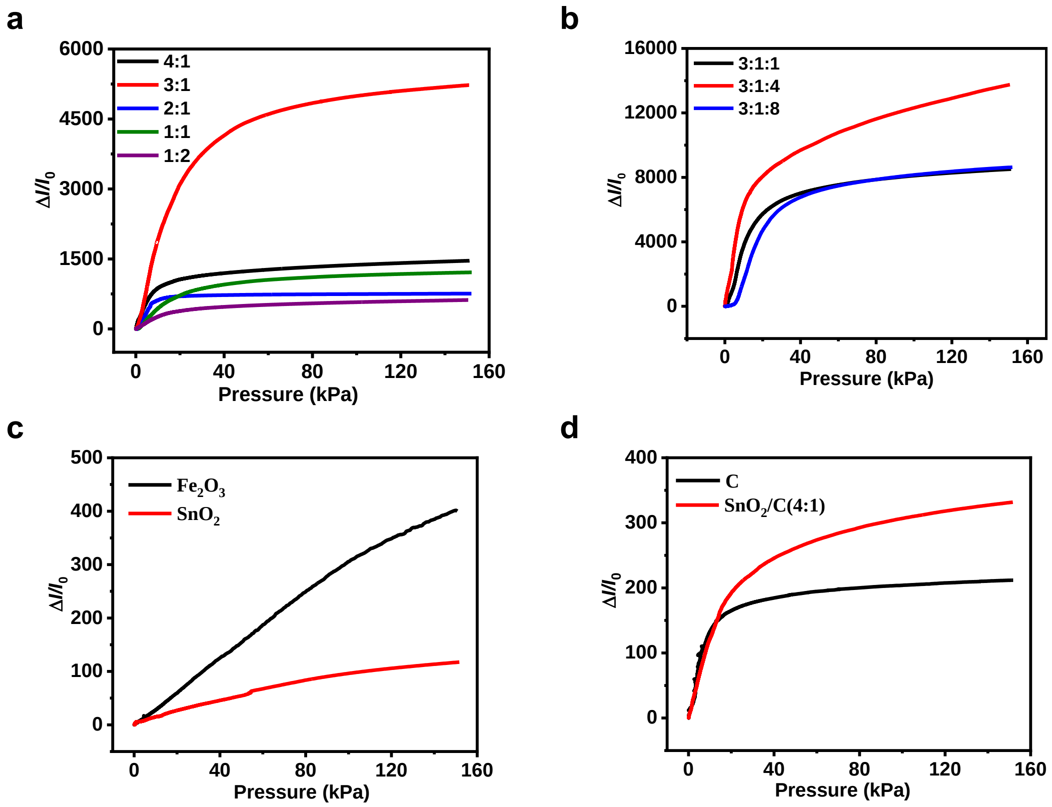

Supplementary Figure 4. Pressure-sensing characterizations. **a** Relative current change for the applied pressure, where the curves represent the different mass ratios of Fe_2O_3/C (4:1, 3:1, 2:1, 1:1 and 1:2), **b** The different mass ratios of Fe_2O_3/C@SnO_2 (3:1:1, 3:1:4 and 3:1:8), **c** Fe_2O_3 and SnO_2, **d** C and the mass of SnO_2@C 4:1.

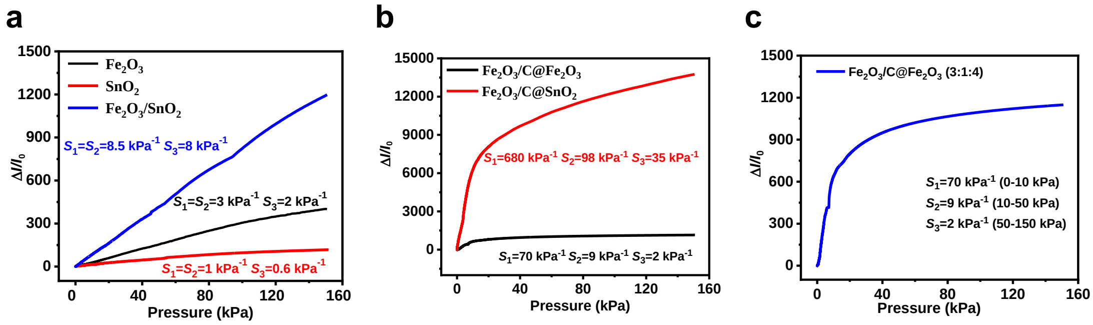

Supplementary Figure 5. Pressure-sensing characterizations. **a** The sensitivity of Fe_2O_3, SnO_2, and Fe_2O_3/SnO_2 pressure sensor. **b** The sensitivity of Fe_2O_3/C@ Fe_2O_3 (3:1:4) and Fe_2O_3/C@ SnO_2 (3:1:4) pressure sensor. **c** The sensitivity of Fe_2O_3/C@ Fe_2O_3 with the mass of ratio of 3:1:4 based sensors.

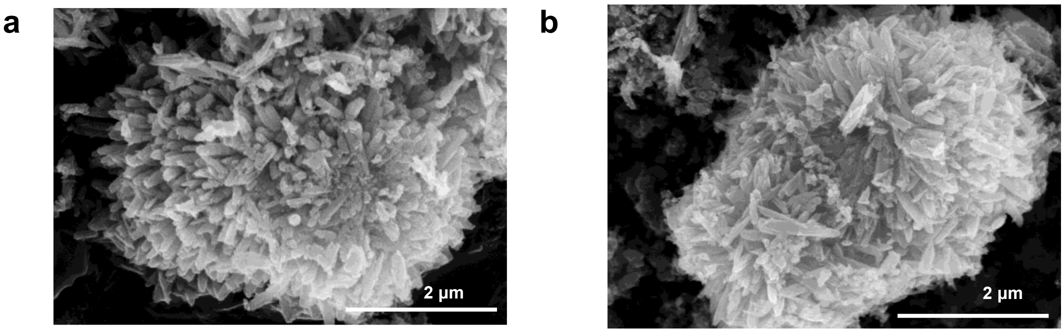

Supplementary Figure 6. SEM images of Fe_2O_3/C@ Fe_2O_3 (3:1:4)

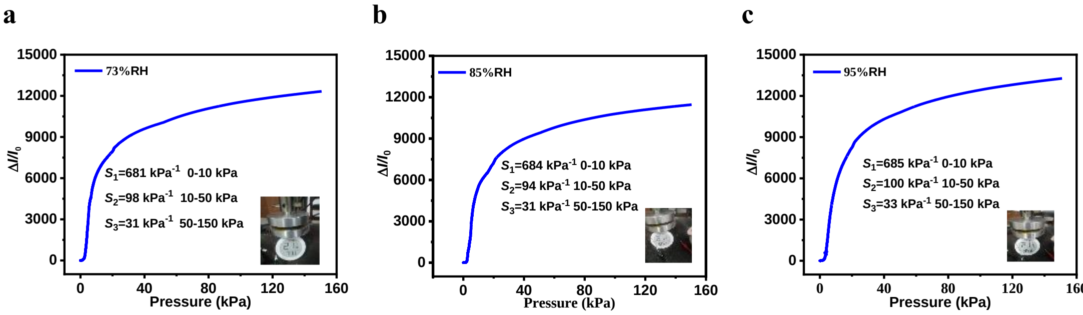

Supplementary Figure 7. Sensing performance under different relative humidity. Sensitivities of Fe_2O_3/C@SnO_2 (3:1:4) pressure sensor under the RH of **a** 73%, **b** 85% and **c** 95% at room temperature.

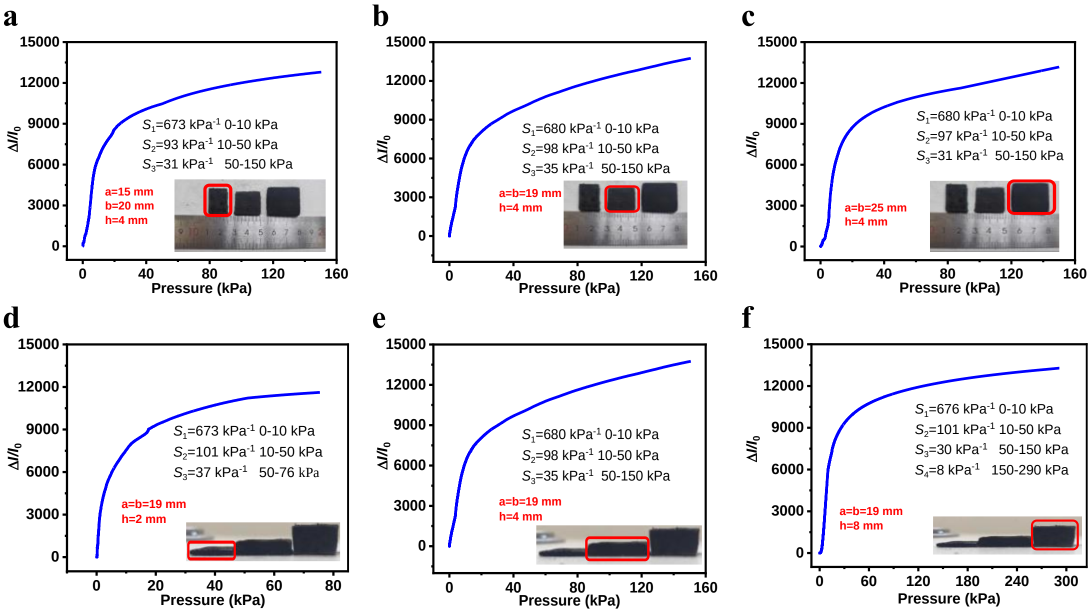

Supplementary Figure 8. Sensing performance under different areas and thickness. The current ratio variation with pressure of the Fe_2O_3/C@SnO_2(3:1:4) sponge **a-c** with a sizes of 15×20 mm, 19×19 mm and 25×25 mm, **d-f** thickness of 2 mm, 4 mm and 8 mm, respectively.

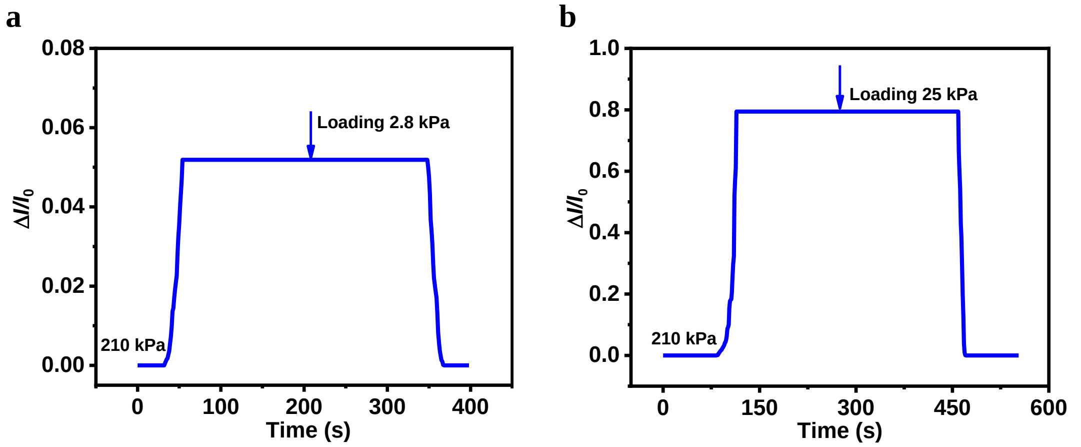

Supplementary Figure 9. Detection of micro pressure under loading pressures. **a** 2.8 kPa and **b** 50 kPa.

Supplementary Table 1 Sensitivity summary of pressure sensor in this work

Materials	Sensitivity(kPa^{-1}) (0-10 kPa)	Sensitivity (kPa^{-1}) (10-50 kPa)	Sensitivity (kPa^{-1}) (50-150 kPa)
Fe_2O_3	3	3	2
SnO_2	1	1	0.60
C	15	0.88	0.20
Fe_2O_3/C (4:1)	88	7	2.30
Fe_2O_3/C (3:1)	203	44	6
Fe_2O_3/C (2:1)	76	9	3.90
Fe_2O_3/C (1:1)	47	8	1.27
Fe_2O_3/C (1:2)	28	3	1.02
SnO_2/C (4:1)	13	2.4	0.7
Fe_2O_3/C@SnO_2 (3:1:1)	420	80	12
Fe_2O_3/C@SnO_2 (3:1:4)	680	98	35
Fe_2O_3/C@SnO_2 (3:1:8)	314	65	11

Supplementary Table 2 Sensitivity summary of pressure sensor in reference

Materials	Nanostructure	Sensitivity (kPa^{-1})	Reference
ZnO/PDMS	Sea urchin-like	75 - 121 (0 - 200 Pa)	1
Au/Ag/PU	Sea urchin-like	2.46 (0 - 1 kPa)<br>0.52 (1 -8.2 kPa)	2
C/PDMS	Sea urchin-like	263 at 1 Pa	3
Mxene/Reduced Graphene Oxide Aerogel	Naosheets	0.55 (23 - 982 Pa)<br>3.81 (982Pa - 10 kPa)<br>2.52 (10 - 30 kPa)	4
Carbon Black@Polyurethane Sponge	Nanosheets	0.068 (0-2 kPa)<br>0.023 (2-10 kPa)<br>0.036 (10-16 kPa)	5
MXene/Sponge	Nanosheets	147 (0-5 kPa)<br>442 (5-20 kPa)	6
Graphene/Eco-flex	Triode-mimicking	4.68 (0-150 kPa)<br>11.09 (150-200 kPa)	7
CNT/cotton textile	Nanowires	14.4 (0-3.5 kPa)<br>7 (3.5-15 kPa)	8
MXene/tissue paper	Nanosheets	0.55 (0-3 kPa)<br>3.81 (3-10 kPa)	9
Fe_2O_3/C@SnO_2/Sponge	Sea urchin-like	680 (0-10 kPa)<br>98 (10-50 kPa)<br>35 (5-150 kPa)	In this work

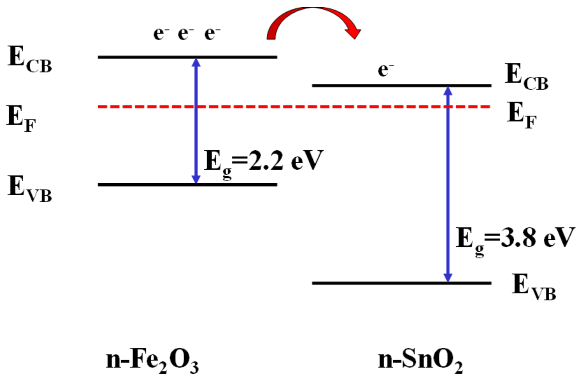

Supplementary Figure 10. Schematic illustration of formed Fe_2O_3-SnO_2 n-n heterojunction. E_{CB}: conduction band edge energy, E_{VB}: valence band edge energy, E_{gap}: band gap energy, E_F: fermi energy.

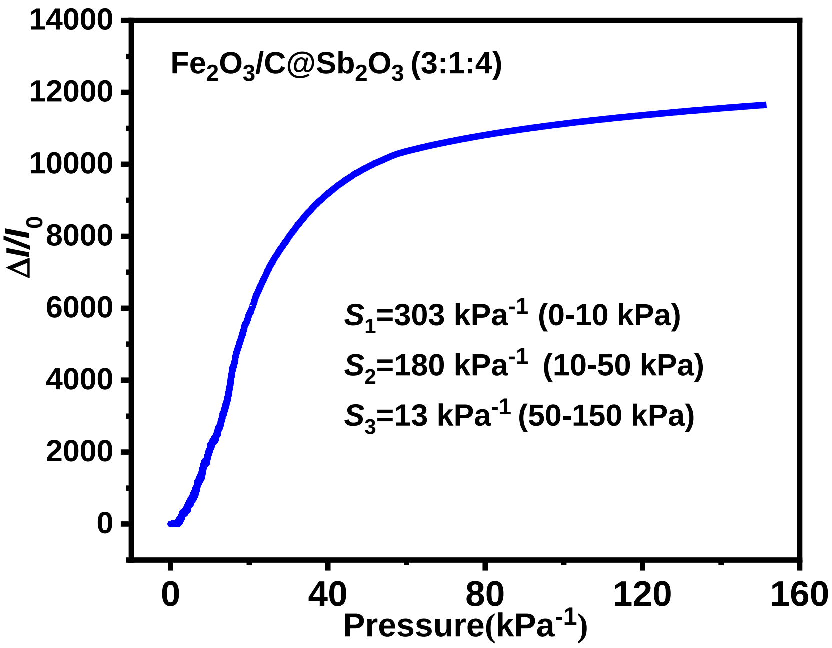

Supplementary Figure 11. Change of current with pressure increases for Fe_2O_3/C@Sb_2O_3.

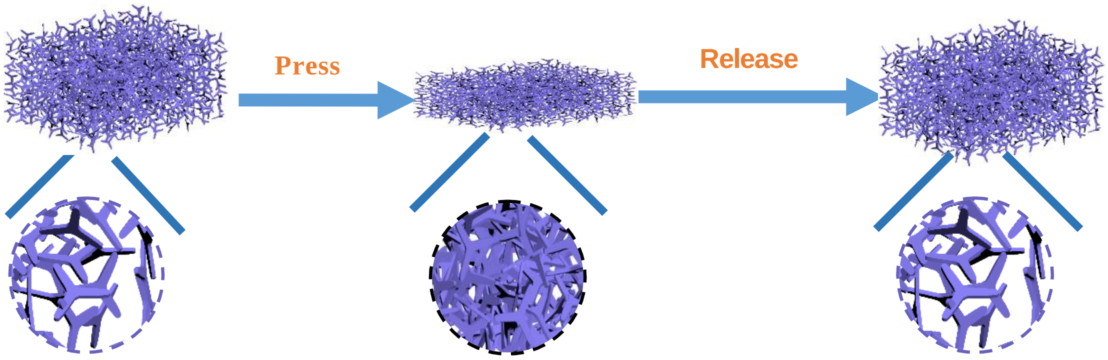

Supplementary Figure 12. The schematic pressure-sensing models of the sponge.

Reference

1. Yin, B., Liu, X., Gao, H., Fu, T., & Yao, J. Bioinspired and bristled microparticles for ultrasensitive pressure and strain sensors. *Nat. Commun.* **9**, 1-8 (2018).
2. Lee, D. et al. Highly sensitive, transparent, and durable pressure sensors based on sea-urchin shaped metal nanoparticles. *Adv. Mater.* **28**, 9364-9369 (2016).
3. Shi, L. et al. Quantum effect-based flexible and transparent pressure sensors with ultrahigh sensitivity and sensing density. *Nat. Commun.* **11**,1-9 (2020).
4. Ma, Y. et al. 3D synergistical MXene/reduced graphen oxide aerogel for a piezoresistive sensor. *ACS Nano,* **12**, 3209-3216 (2018).
5. Wu, X. et al. Large-Area Compliant, Low-Cost, and Versatile Pressure-Sensing Platform Based on Microcrack-Designed Carbon Black@Polyurethane Sponge for Human-Machine Interfacing. *Adv. Funct. Mater.* **26**, 6246-6256 (2016).
6. Yang, Y. et al. 3D hybrid porous Mxene-sponge network and its application in piezoresistive sensor. *Nano Energy.* **50**, 79-87 (2018).
7. Wu, Q. et al. Triode-mimicking graphene pressure sensor with positive resistance variation for physiology and motion monitoring. *ACS Nano.* **14**, 10104-10110 (2020).
8. Liu, M. et al. Large-Area All-Textile Pressure Sensors for Monitoring Human Motion and Physiological Signals. *Adv. Mater.* **29**,701-709 (2017).
9. Ying, et al. A Wearable Transient Pressure Sensor Made with MXene Nanosheets for Sensitive Broad-Range Human-Machine Interfacing. *Nano lett.* **19**, 1143-1150 (2019).
